# Banal Radicalism: Free Spaces and the Routinization of Radical Practices in Far‐Right Movements

**DOI:** 10.1111/1468-4446.13213

**Published:** 2025-04-08

**Authors:** Oded Marom

**Affiliations:** ^1^ University of Southern California Los Angeles California USA

**Keywords:** far‐right, free spaces, libertarians, prefigurative politics, radicalization, social movements

## Abstract

How do free spaces become radicalizing spaces? Studies of far‐right radicalism have highlighted the role of insulated movement spaces in radicalizing their members. In these spaces, participants can flaunt their radical ideas and infuse them into everyday practices, forming these ideas into comprehensive and resilient worldviews. However, the salience of radical ideas in free spaces has also been found to be inconsistent and rare. This contrast makes it unclear when and how exactly free spaces contribute to the spread and persistence of radical ideas. Drawing on a 4‐year ethnographic study of a radical right‐wing libertarian movement in the US, this study shows how activists both highlight and downplay radical ideas creatively to solve situationally emergent challenges of coordinating action. Thus, while the movement's free spaces created circumstances that imbued some everyday mundane practices with radical political significance, they also facilitated an opposite process: they created conditions that obscured or even undermined the political meaning of otherwise radical practices. As I argue, rather than stifling the spread of radical ideas, this banalization of radical practices is a critical component of the radicalization process itself, allowing activists to coordinate radical action among a diverse group of people and across varying situations. In this way, free spaces contribute to the coordination of radical action, even among participants who do not necessarily express radical political motivations. Thus, the findings show how people's motivations for radical action are often articulated in the moment, in response to specific situations and the challenges they present.

## 1 Introduction

The past 2 decades have brought a global rise in the popularity and visibility of radical right‐wing social movements that promote racist sentiments and mistrust in liberal democracy, often touting allegations of deep‐rooted conspiracies among government and global elites. This trend has been a cause of academic and public concern, as some of these movements inspire their participants to amass firearms, challenge local, state, and federal authorities, and, in extreme cases, organize collective acts of intimidation and violence. To address this concern, social scientists have begun paying increasing attention to the ongoing practices in these movements that enable them to sustain and spread their ideas despite suffering stigmatization and backlash from broader society (Abbas 2023; Blee et al. 2024).

In particular, considerable attention has been given to the role of movements' “free spaces” (Polletta 1999) in recruitment, organization, and radicalization of far‐right activists (Futrell and Simi 2004; Futrell et al. 2018; Luger 2022; Miller‐Idriss 2020). In these spaces, movement participants form new relationships with other like‐minded people, feeling free to flaunt the radical ideas that they are otherwise forced to hide, and to infuse these ideas into everyday practices such as cooking, gardening, and even parenting (Simi et al. 2016). In the process, members learn to translate their everyday implicit prejudices and transgressive beliefs into comprehensive worldviews that can remain obstinate in the face of counterarguments or critique (Blee 2003; Simi and Futrell 2010).

However, as other studies, particularly of left‐leaning free spaces, have shown, the relations between activists' practices and ideals in these spaces are often varied and complex (Maeckelbergh 2009; Yates 2015b). Participants use free spaces to challenge, debate, and experiment with different practices and ideological positions, and different activists often imbue similar practices with varied meanings. And while some scholars have highlighted how free spaces' participants charge their everyday practices with political significance (Yates 2015a), others have shown that this is the exception rather than the rule. For the most part, free spaces are sustained by routine, mundane, and repetitive tasks involving little to no ideological reflection (Glass 2010).

Radical ideals, therefore, are not consistently salient in free spaces. Rather, they only become salient at certain times and in different ways. This raises important questions for those interested in the radicalizing effect of free spaces, mainly *when and how free spaces actually contribute to the persistence and spread of radical ideas.* Specifically, we should ask, when exactly do participants in free spaces invoke their radical ideas? How do they decide which practices carry radical meanings and which should be treated as mundane tasks? And what happens in free spaces when radical practices become routine work? In other words, when and how do “free spaces” become “radicalizing spaces”?

In this article, I argue that to properly understand when and how free spaces facilitate the dissemination and endurance of radical ideas, we need to examine how activists in these spaces creatively politicize and depoliticize their radical practices in response to emerging challenges of coordinating action. Drawing on a 4‐year ethnographic study of a radical right‐wing libertarian movement in the US, this article shows how activists responded to varying interactional contexts by highlighting or downplaying the ideological significance of their practices, to solve *situationally relevant challenges* and enable smooth coordination. Thus, while the movement's free spaces created circumstances that imbued some everyday mundane practices with radical political significance, these spaces also facilitated an opposite process: they created conditions that obscured or even undermined the political meaning of otherwise radical practices.

In this way, free spaces facilitate what I term *banal radicalism*,^1^ everyday habits that reproduce ideological representations and practices while toning down explicit expressions of the group's radical political agenda. As I argue, rather than stifling the spread of radical ideas in the movement, banal radicalism is a critical component of the process of radicalization itself. Downplaying their practices' radical meaning allows activists to creatively respond to emerging problems and adjust these practices to the changing needs of their everyday lives. By moving between banal and explicit representations of their radical practices, activists manage to coordinate these practices among a diverse group of people and across varying situations. Thus, banal radicalism works in tandem with more explicit forms of radicalism to facilitate the resilience and spread of radical ideological practices over time.

To illustrate this, I show how right‐wing activists reacted differently to varying coordination challenges in three kinds of situations. First, I show how, in the movement's free spaces, the coordination of well‐established, routinized alternative practices often took the form of “training sessions.” In such sessions, participants were predominantly concerned with *practical challenges* and focused on disseminating the necessary skills needed to successfully carry out and integrate these practices into their everyday lives. In the process, the practices' radical meanings were downplayed and interactants treated them in nonideological, banal terms. Next, I show how, outside of training sessions, coordinating less‐established practices often involved various *practical and ideological concerns* *and disagreements*. In such situations, activists responded to emerging challenges by creatively downplaying and highlighting the radical meaning of the same practice, depending on people's specific concerns and the nature of the disagreement. Finally, I examine how a unique type of situation—one involving publicly staged interactions—prompted activists to focus on *ideological challenges*, tying their practices to deep political questions about their group's radical goals and shared utopian vision. Such staged interactions prompted participants to highlight the radical meaning of their practices, but they were also logistically complex and relatively costly. As I suggest, this may explain why explicit ideological performances are the exception, rather than the rule, in movements' free spaces.

These findings illustrate how the radical meaning of a practice does not depend entirely on the practice itself, but also on the social situation in which it is negotiated and exercised. Rather than being a feature of any particular practice, radical political meaning is also a product of the challenges collective actors face as they try to coordinate action, of their interpretation of these challenges, and of their attempts to solve them together.

This situational nature of radical meanings is particularly important considering recent developments in our understanding of the relationship between motivation and action. As contemporary pragmatist sociologists argue, actors' motivations are articulated within specific situations through their collective efforts to find solutions to emerging problems (Flores and Gross 2022; Jansen 2016; Whitford 2002). Similarly, cultural and cognitive sociology studies have shown that motivations and moral rationales for action can often follow rather than precede behavior (Luft 2023; Vaisey 2009). These claims align with studies of right‐wing extremism, which show that people often participate in radical right‐wing practices because of the relationships they establish with other practitioners, and only later do they adopt ideological beliefs that justify their behavior (Blee et al. 2024).

Thus, participation in radical practices can often precede or even lead to radical motivations. Understanding the process of banal radicalism can help us see how free spaces allow for ongoing coordination of radical political action, even among participants who do not express radical political motivations. Moreover, this process directs our attention to how motivations and justifications for radical action are not necessarily pregiven but can develop out of specific situations and the challenges they raise. Banal radicalism highlights the way movements' practices still play a role in the radicalization of their members, even when these practices do not include explicit displays of the group's radical ideology and political goals.

### 1.1 Radicalizing Spaces

“Free spaces” are social settings within a movement that are removed from the direct control and surveillance of dominant groups. In those spaces, activists are free to develop cultural challenges to the predominant social and political order and to try out alternative institutions and practices that they envision in their ideal version of society (Polletta 1999). This has made free spaces particularly interesting for scholars studying right‐wing radicalism. As these scholars show, far‐right activists use a variety of settings secluded from mainstream society to meet and safely express their beliefs without risking repercussions (Davis and Kettrey 2023; Miller‐Idriss 2020). Settings such as online discussion groups, fight‐clubs, music venues, outdoor festivals, private communities, and even family households serve as spaces where members can openly discuss, practice, and develop their radical ideas. In these spaces, members often participate in collective practices that solidify their commitments to the group and stabilize their extremist identities (Latif et al. 2018; Simi and Futrell 2010). In the process, people learn to connect their everyday experiences with the movement's principles and ideology and to articulate their underdeveloped and implicit transgressive beliefs as comprehensive interpretive schemas that render them more resilient to challenge and critique (Blee 2003).

As various studies of far‐right movements show, private settings such as the household, informal social gatherings, or Bible study groups become templates for members of how interpersonal and even intimate relationships ought to be in their utopian society (Futrell and Simi 2004). In these spaces, activists imbue otherwise innocuous activities, like homeschooling or child discipline, with racist fantasies and oppositional movement convictions. Politicizing everyday activities (such as parenting), allows activists to integrate and align their potentially conflicting personal, social, and political identities (Simi et al. 2016). Similarly, far‐right activists often adopt politically motivated consumption habits, such as exclusive commerce with like‐minded service providers as a means to support fellow ideologues or avoid supporting minority groups (Blee 2003). For others, certain lifestyle choices are infused with extremist ideas. For example, far‐right online cooking shows or guides for growing and catching one's own food are commonly peppered with radical anti‐immigration, antigovernment, antisemitic, and similar messages (Miller‐Idriss 2020). Infusing radical ideas into everyday practices normalizes these ideas and connects them to activists' lives in palpable ways, formulating their activist identities and sense of commitment to the cause.

Hence, these studies show how imbuing everyday practices with explicit radical messaging makes movements' ideologies a salient and meaningful part of their members' lives, playing a central role in their radicalization. However, the relations between practices and political ideals in free spaces are far from uniform. This conclusion is well established in studies of left‐leaning movements' free spaces.

Since the 1970s, students of anarcho‐communist and similar leftist movements have questioned activists' efforts to develop alternative practices emulating the cultural and institutional futures that their movements envisioned.^2^ In particular, critics have debated the nature of relations between these practices and the ultimate political ideals they aimed to realize (Boggs 1977; Lipset and Altbach 1969). What began as a debate about political efficacy developed over time into discrepant descriptions in the literature of the political meanings and ideological motivations behind alternative practices. Some (e.g., Breines 1989; Epstein 1991) have described alternative practices as a form of propaganda that activists perform to advocate for and advance political change, whereas others (e.g., Graeber 2002, 2009) have described them as a set of moral practices that activists adopt as a good unto themselves (see also Gordon 2018; Swain 2019; Yates 2015b).

As empirical studies have shown (Maeckelbergh 2009; Yates 2015b), this discrepancy relates to the fact that activists themselves express various motivations for their practices, imbuing them with diverse and often inconsistent political meanings. In fact, the political meaning of many practices only becomes visible in special circumstances, such as in interviews, or when activists perform the practices for outsiders or present them in the movement's literature (Yates 2015a). Typically, many of activists' everyday activities are treated as “maintenance” activities to sustain their shared spaces, and their political meaning only becomes salient on rare occasions when people stop what they are doing to actively engage in shared political reflection (Glass 2010).

As Luger (2022) has shown in his study of everyday spaces of the American far‐right, this fluidity allows certain mainstream spaces and activities to have extreme and even violent potential, where radical mobilization often “emerges” or “erupts” out of daily activities like watersports, cocktail parties, group workouts, and religious ceremonies. Drawing on Hardt and Negri's concept of “the swarm,” Luger suggests that these eruptions are not random but born out of collective and decentralized techniques of problem‐solving in the group. What remains unanswered is *what problems* and *what techniques* lead to far‐right radicalization, and *how*? As I will now show, to answer these questions, we should draw upon tools from the contemporary pragmatist approach in sociology.

### 1.2 Coordination as Problem Solving: A Pragmatist Analytical Approach

To understand how activists' everyday habits facilitate radicalization processes, this study adopts a pragmatist approach to analyzing collective action. Rather than thinking about action in utilitarian terms in which actors rationally devise the means to achieve their predetermined ends, a pragmatist approach gives particular attention to the situations in which people try to coordinate action and the way situational conditions shape their understanding of the problems they face and of appropriate solutions (Joas 1996; Whitford 2002). Therefore, to understand how people coordinate collective action, pragmatist sociologists examine how people interpret their situations, develop shared understandings of emerging challenges, and devise available paths forward (Elfakhani 2022; Gross 2009; Jansen 2016). In particular, a pragmatist approach pays special attention to the ways actors treat certain problems as private or immediate difficulties that require ad hoc solutions and other problems as broad general issues that require a political response (Boltanski and Thévenot 1991; Eliasoph and Lichterman 2010; Thévenot 2014).

Importantly, such “specifying” and “generalizing” of problems should be understood as a practical achievement. Drawing on Charles S. Peirce’s semiotic approach, Tavory and Timmermans (2022) showed how actors move from specific to general scopes of interpretation as part of their collective efforts to construct a shared understanding of the world in varying social situations. While actors enter social situations with preexisting interpretational habits, they are also able to creatively alter their interpretations according to the social situation and the other actors involved. Different situations and different participants pose different challenges to one's interpretations, and people can respond and adjust accordingly. As they work to negotiate shared meanings, actors “upshift” and “downshift” their discussions, moving from the specific to the general and vice versa, in an attempt to generate agreement, sway others, and promote their own goals.

This movement has meaningful implications for how and when politics becomes a meaningful part of people's everyday lives. When faced with a problem or a conflict in their lives, actors may view it as a unique, isolated incident, or as a local variation of a broader social issue. Thus, as actors upshift a conflict, treating a local occurrence as a representative of a social type, they effectively construct it as a social problem, giving it political significance. Similarly, actors may downshift a problem by treating a conflict as a discrete local incident without meaningful social implications. This latter tactic sometimes serves as a “weapon of the weak” as it allows actors to disarm stereotypes or avoid touchy political conversations, but it can also serve to dismiss claims about discrimination and silence publicly‐minded arguments about the common good (Eliasoph 1998; Timmermans and Tavory 2020).

Political meanings, therefore, are products of actors' shared efforts to collaborate and reach agreements; efforts that are always made within specific situations and in response to emerging situational challenges. As I illustrate below, this approach allows us to examine how activists construct and deconstruct political meanings as part of their ongoing processes of coordinating action, and how both construction and deconstruction can play a role in the reproduction of radical ideological representations and practices over time.

### 1.3 Radicalism, Populism, and the American Far‐Right

Before moving forward, it is worth clarifying what we mean when discussing far‐right radicalism. The term far‐right has been used to describe diverse phenomena, from violent neo‐Nazi terrorism in Europe to the Jewish nationalist coalition of prime minister Benjamin Netanyahu in Israel. While these phenomena differ in many ways, what brings them together is their hostility toward liberal democracy, and particularly toward non‐majoritarian state institutions that allow elite actors to curb the will of “the people” in the name of minority rights, rule of law, and separation of powers (Mudde 2019; Rydgren 2018; Wodak 2020).^3^

The critique of elites' excessive power in government traditionally comes from both the left and the right. However, while left‐wing critique is rooted in socialist, internationalist sentiments about universal equality, right‐wing arguments describe inequalities between people (be those economic, racial, ethnic, and so on) as natural and positive, and their critique is directed at government elites' attempt to undermine and erase these differences. Such right‐wing populism proclaims to oppose a homogeneous and corrupt elite in the name of the “pure people,” a category that can (and often does) exclude ethnic, racial, and religious minorities, but also economic elites and educated intellectuals (Pelinka 2013; Mudde 2019).

In the United States, these populist sentiments have been uniquely inflamed by the objection to administrative government coming from white Christian Nationalists who reject racial justice programs, Republicans who advocate for expanded powers to the presidency, and extreme libertarians who seek to minimize government intervention in economic and social affairs. While their goals may differ, these movements' shared crusade against government bureaucracy has contributed to the mainstreaming of “deep state” conspiracy theories and support for strong leaders who presumably better represent the people's will (Hanson and Kopstein 2024). As these sentiments have become more widespread, they have begun to motivate diverse forms of far‐right political action, from support of the Tea Party movement to participation in the January 6 attack on the Capitol Building (Hochschild 2018; Valayden et al. 2024).

Such populist, anti‐government sentiments correspond with similar far‐right programs across the world. In Europe, several countries have witnessed a coordinated attack on professional government elites, bolstered by popular resentment against the EU and its bureaucratic apparatus. Most notably, in Hungary, Prime Minister Viktor Urbán has leveraged this resentment to undermine the independence of non‐majoritarian institutions and to purposely transform the country into an illiberal state (Mudde 2019; Hanson and Kopstein 2024). In other countries, such as Israel, far‐right populism has been quite literally imported from the United States, as Jewish‐American immigrants established civil‐society organizations in Israel to spread nationalist and libertarian ideals and reshape the country (Segal and Greenspan 2024).

In what follows, I will examine a far‐right movement representing the libertarian leg of this populist stool and show how its members coordinate their radical practices in a way that enables their normalization and longevity.

## 2 Case Study and Methodology

This study is based on 4 years of participant observation with the “Free State Project” (FSP), a US‐based right‐wing movement seeking to establish a utopian libertarian community in the state of New Hampshire, take over local government, and potentially secede from the Union. The movement began organizing in 2001 and today has approximately 5000 members, colloquially called “Free Staters” (or “FSers”).

The United States has a long tradition of libertarian utopianism. During the 19th century, the country witnessed the emergence of a multitude of intentional communities seeking political, spiritual, and social liberation with varying success (Kanter 1972). The FSP could be well described as a contemporary iteration of this tradition but, at the same time, it should also be understood within the context of the modern American libertarian movement.

In the 1960s–1980s, the rise of the American New Left and Hippie movements brought along a series of experimental independent communities that advocated for alternative ways of organizing public life and challenged traditional institutions (Berger 1981; Breines 1989; Epstein 1991). Animated by the era's anarchist and anti‐government sentiments, young members of the American right began segmenting from the Conservative movement and rallying around a wholesale rejection of government involvement in any aspect of economic and personal affairs (Klatch 1999). This right‐wing version of libertarianism found inspiration in laissez‐faire market idealists like Ludwig von Mises, Friedrich Hayek, Milton Friedman, and Ayn Rand, and advocated for causes like the abolition of income tax and the end of the welfare state (Doherty 2007). Members of this newly formed libertarian movement coalesced within various political institutions such as the Cato Institute and the American Libertarian Party. However, some radical groups, like the FSP, opted for more extreme approaches, seeking to establish radical libertarian communities that could realize the movement's ideals through direct action and other anti‐institutional tactics.

Like other radical right movements (see Miller‐Idriss 2020), FSers uphold deep antigovernment and anti‐establishment ideals. Alongside their declared goal of gaining independence from the federal government, activists regularly challenge local authorities and often actively obstruct the work of state and government agents. FSers regularly protest and disrupt police sobriety checkpoints or harass local parking meter attendants to prevent them from enforcing city parking ordinances. Some opt for more institutionalized tactics and run for local political offices. Once elected, FSers work to slash funds for government services such as public education, police, and even fire departments (Dietsch 2023; Hongoltz‐Hetling 2020). The movement's resentment toward government institutions is bolstered by commonly shared conspiracy theories, from the US government's responsibility for the 2001 attack on the World Trade Center to the existence of hidden international forces controlling the American government from the shadows.^4^ FSers are also great advocates for popular gun ownership, and members commonly own and carry firearms for protection or public display.

Like other far‐right movements, the FSP is predominantly white. However—and importantly—unlike other far‐right movements, the FSP does not formally uphold exclusionary racist beliefs and is generally nonviolent. Still, local pro‐democracy watchdog organizations have argued that shared anti‐government goals have brought the FSP closer with white‐supremacist and militia groups in the state, and that some FSers have gone so far as to threaten violence against politicians (Dietsch 2023).

Additionally, the FSP differs from some other far‐right movements by having no clear position on questions of gender. Whereas the far‐right has been identified with advocating for traditional gender roles and especially a celebration of hyper‐masculinity (Miller‐Idriss 2020; Luger 2022), the FSP expresses a more open approach to gender relations. The movement includes some openly LGBTQ members, and some FSers live in nontraditional family households. Still, like the broader American libertarian movement, the FSP is predominantly a male movement. Movement spaces are thus mostly occupied by straight men who tend to exhibit traditional masculinity. In terms of class, the FSP includes some middle‐class professionals alongside professional artisans and day‐laborers, some of whom can be described as working poor.

FSers live in several communities across New Hampshire, and they meet regularly in various movement spaces, including outdoor festivals, private clubhouses, independent farms, and members' private homes. In these spaces, members experiment with various alternative practices that simulate the conditions for and reality of a government‐less society. I conducted participant observation with members in these spaces from winter 2016 to summer 2019, presenting myself as a sociologist interested in the group's unique form of political organizing. I joined the group's political meetings and events, attended their annual “campouts,” visited their community market days, attended their weddings and vigils, and socialized with them regularly in their community “clubhouses,” private homes, and other social gatherings. In total, I conducted over 600 h of participant observation, producing over 800 pages of field notes and 36 h of audio‐recorded conversations.

To understand how activists impart varying meanings into their everyday practices, my analytical approach focused on moments when activists tried to coordinate their radical practices, and particularly on their attempts to articulate the meaning of their practices and the problems they aim to solve. To do this, I coded my field‐notes in a two‐cycle process. First, I used topic coding, noting each passage's subject matter. Then, I focused on passages where activists tried to coordinate their varying practices and coded these passages line‐by‐line to identify patterned differences in how activists described their difficulties, disagreements, or proposed solutions. Specifically, I focused on the differences between times when actors described problems and solutions in practical or personal terms and times when they described them as political issues that concern the group or its mission. This allowed me to examine how varying situations pose different challenges for coordination, prompting activists to apply different interpretational scopes and to upshift or downshift their discussion and the meaning of their practices according to situational needs.

### 2.1 Studying the Radical Right: Motivations, Rapport, and Ethical Considerations

Researchers of far‐right movements have highlighted the risk associated with conducting academic work that rationalizes and publicizes far‐right views and practices, as it may legitimize and empower far‐right activists and play a part in advancing their causes (Blee 2003). Accordingly, some studies take a normative position that “judges” their subjects and highlights the deviant and abnormal nature of their politics (Bauer et al. 2024). Yet by the time I entered the field in late‐2016, far‐right politics could hardly be described as “abnormal.” Rather, as the political ascendence, and then election of President Donald Trump powerfully signified, far‐right politics in the US have become mainstream (Mudde 2019). Therefore, rather than judgment, my motivation for this study was to understand how people become radicalized into far‐right causes, integrating these causes into their lives in a way that allows them to become “mainstream” and “normal.”

The FSP's desire to establish a right‐wing libertarian community made it an ideal candidate for exploring this question. However, entering the field proved to be a protracted process. My initial contact with the FSP was through a post on the movement's Facebook page, where I presented myself and expressed my interest in interviewing members. Although I did not receive much response, one well‐known figure in the group replied, saying that he was planning to visit my city soon and would be willing to meet me for coffee. After we met, this member shared his positive impression with me on the group's Facebook page and recommended that others accept my invitations to be interviewed. As in other ethnographies of the far‐right (Fangen 2020; Ramalingam 2020), such an endorsement from a leading figure made other members keener to respond to me and facilitated my entry into the group.

Unexpectedly, my ethnicity proved also valuable here. When I posted my introduction, two prominent Jewish members of the group inferred from my name that I was also Jewish and offered to host me in their homes if I came to New Hampshire. Although I did not end up staying with them, these members facilitated my early visits, showing me around and introducing me to other FSers. As other ethnographers have also found (see Ramalingam 2020; Deodhar 2022), being an ethnic minority can present both challenges and opportunities when studying the far right. In my case, being Jewish allowed me to find a commonality with some central figures that helped overcome our ideological differences and assuaged suspicion.

That said, other FSers remained highly suspicious of me for a long time. As in other ethnographies of the far‐right (Fangen 2020; Simi and Futrell 2010), FSers suspected that I might be a government agent trying to infiltrate the group. Some confronted me directly about this while others regularly presented me with ideological “purity tests” to examine my feelings about the group's causes and ideals. Rather than trying to “give the right answer,” I decided to answer these tests honestly and be straightforward about my ideological differences with the group. Ironically, this approach seemed to improve my credibility and appeal. Some FSers enjoyed the opportunity to debate ideology, and others simply appreciated my candor. It probably also helped that I repeatedly clarified to members that my research interest was not their political views, but their coordination mechanisms and interactional processes.

Rather than sharing politics, I connected with my subjects through shared experiences over time. I often volunteered to help members with various tasks: I helped them move; handed out fliers with them; accompanied them to demonstrations; and even volunteered to work in the kitchen at a wedding of two FSers. Through these shared experiences, I developed personal connections with members, and our relationships became less formal and more friendly with time.

Naturally, some FSers remained suspicious of me to the very end, turning eerily quiet or carefully weighing their words whenever they noticed my presence. However, I never faced any threats or direct confrontations. That said, the fact that some subjects preferred not to collaborate with my research meant that certain aspects of the movement remained unavailable to me. As such, this study should not be taken as a comprehensive portrait of the FSP, but as a focused analysis of specific movement practices and their importance for far‐right radicalization.

Still, for the most part, being male and white helped me blend into the group, and I found that it sometimes required special effort on my behalf to clarify that I was not in fact a fellow member, but rather a researcher studying the group, to make sure that I satisfied the IRB's ethical guidelines and obtained people's consent. In this sense, my ethical considerations were not only about the implications of studying the far right but also concerned with the safety and fair representation of my subjects. For this reason, I changed all names in my field‐notes, omitted people who asked not to be documented, and limited my report to observations that would not put my subjects at risk, legal, or otherwise. Finally, during my time in the field, I discussed my findings with my subjects and later shared my published articles with the group. This not only helped assuage people's concerns but hopefully also lessened their suspicion and resentment toward academics and liberal intellectuals in general, increasing the plausibility of more similar research in the future (see Deodhar 2022, 553–4).

## 3 Findings

### 3.1 Training Sessions: Banal Coordination of Established Radical Practices

FSers widely reject the right of government to regulate and tax any form of transaction or production. In their free spaces, members implement and experiment with various alternative practices that enable them to escape government supervision and simulate their ideal vision of a libertarian society. However, as these radical practices become routinized in people's everyday lives, members become increasingly concerned with the practical challenges involved in carrying the practices out successfully, and the coordination of these practices commonly takes the form of practical training, where activists teach each other the necessary skills to overcome these challenges. In such social settings, radical practices take on a pedestrian quality, and their political revolutionary meaning is downplayed or utterly ignored. In this way, these “training sessions” have the paradoxical effect of reproducing and disseminating ideologically motivated practices in the movement while simultaneously toning down those ideological motivations.

Thus, independent farmers and urban gardeners in the FSP commonly cultivate crops and livestock in their backyards and use generators and solar panels to achieve self‐sustainability and independence from government regulation and oversight. These activists meet with others in local libertarian crash‐pads, clubhouses, and festivals to trade in self‐produced goods and services, modeling their vision of an unregulated free market and supporting the movement's overall capacity to cultivate a lifestyle independent of government supervision. In these spaces, libertarians develop creative solutions to circumvent government regulations and taxation. Many prefer trading in cryptocurrency or precious metals (mainly silver) over government‐issued currency. Others sell “shares” of expected farm yield rather than existing farm products to sidestep USDA regulations on retailed farm goods, creating what could be described as de facto cooperatives.

To coordinate these alternative practices, libertarians must attain a certain measure of shared practical knowledge. Farming in New Hampshire's hard granite soil and cold weather requires specialized knowledge. To trade in cryptocurrency, one must know how to open an electronic wallet, what different coins are worth (or even which coins exist), and which coins are accepted by other traders. In their free spaces, veteran libertarian farmers and cryptocurrency traders share their knowledge and experience with novices in designated workshops, private training, or simply through casual interactions. It is not uncommon to see vendors and other well‐intentioned members assisting a novice customer to set up a Bitcoin account and walking them through the steps of completing their first transaction.

Aimed at integrating newer members into existing practices, these training sessions are very practical, taking people through the steps of getting the task done without stopping to explicate the broader political end these practices are designed to achieve. For example, in a workshop entitled “Grow Your Own Food,” held during a large outdoor libertarian festival, the speakers instructed members on how to choose the best location for different types of crops, how to protect crops from pests, and how to choose fitting crops for their garden. To help the audience learn the proper technique to loosen the soil, one speaker grabbed a pitchfork he had brought with him and demonstrated, on stage, the precise bodily motions of the task. When the talk concluded, audience members stood up and asked for more specific advice on how to grow sweet potatoes or “nice carrots” and how to combine a gardening schedule with a fulltime job.

Interestingly, at no point during the workshop did anyone mention the political reason for growing one's food in the first place. Even when explaining their choice to use a private lab to test their soil for minerals rather than the available government service, the speakers attributed it to the former being faster and not to any desire to avoid government intrusion. Apparently, for the workshop participants, the problem they faced was a practical one—how to grow crops successfully—and the solutions were discussed in terms of the best way to accomplish that.

It is not that FSers saw no political meaning in their self‐sustainability practices or black‐market trade, nor did they treat them as mere “maintenance tasks.” When asked, activists offered various political motivations for their choice to grow their own crops or use alternative currency. Two common explanations were that these alternative practices allowed activists to avoid funding the government through taxes and that they demonstrate to outsiders that the group's utopian vision of an unregulated, stateless, free‐market society is readily achievable, and worthwhile. However, much like in other movements (Yates 2015a), the political meaning of everyday practices became explicit only in distinct moments of reflection or public performance. Within the social context of the training sessions, FSers remained focused on the nuts‐and‐bolts aspects of these practices. The challenge, as they interpreted it in these settings, was the successful accomplishment of a task, and not the political challenge of reforming society. The goals of these coordination efforts, in other words, were short‐term and local. Whatever underlying broad, long‐term goals members may have had, they apparently found them irrelevant to discuss in this type of social interaction.

Thus, training sessions illustrate how the banalization of radical practices plays an important role in their dissemination and reproduction. Rather than being a mere undesired product of routinization, the toning down of the group's radical ideology in these settings allowed activists to focus on practical solutions for successfully executing and integrating these practices into their everyday lives. Toning down ideological discourse in certain social situations enabled the activists to spread the knowledge necessary for successful participation in the movement's radical conduct.

### 3.2 Scope Switching: Politicizing and Depoliticizing Practices to Resolve Coordination Challenges

Not all coordination is simply a matter of following or integrating members into established practices. In some cases, coordination is a matter of establishing new practices or improving existing ones. Unlike established practices, where participants assume there is an accepted way of carrying them out and experts can bestow their knowledge on novices, new, or reimagined practices leave much more room for experimentation, innovation, and debate. In such situations, FSers usually tried out multiple interpretations as they attempted to reach an agreement over the nature of the problem they were trying to solve and its appropriate solutions. This proved useful for coordinating action, as the availability of multiple interpretations enabled actors to switch between them as needed and to imagine the same practice through different scopes, sometimes treating it as a private or practical act and sometimes as part of a grand political project. This fluidity allowed actors to emphasize varying interpretations when interacting with different participants or to abandon interpretations that invoked too much disagreement. Thus, actors could imbue practices with radical political meanings and downplay these political meanings, all during the same discussion, to allow for smooth coordination among the participants. What determined the meaning of the practice, therefore, was not the practice itself but the coordination challenges activists faced and how they attempted to solve them.

This was clearly illustrated when FSers negotiated their alternative approach to parenting and schooling. Like other radical movements, the FSP has taken issue with traditional childcare and education models, including traditional parenting approaches, which foster deference to adult authority. For many libertarians, traditional educational institutions teach children obedience and rule‐following, and public schools specifically are seen as part of an overarching scheme to cultivate an obedient population that the state can easily submit to its will. To shelter their children from these coercive institutions, libertarian parents often look for alternative schooling practices that will nurture and celebrate their children's sense of sovereignty and freedom.

Thus, one summer, during the movement's annual camping event, a group of libertarian parents came together to discuss “alternative childcare options.” As the invitation explained, the meeting's goal was “to start a not‐for‐profit that enables families to make their own connections and find a way to balance work and life outside of conventional childcare centers.” Stacy, the organizer, asked the dozen‐or‐so participants to introduce themselves and then described her motivation for having this meeting. As she explained, she and her husband had struggled with offering their children proper care while holding steady jobs. They had tried a couple of daycares but noticed that their kids came home exhausted, and their attitudes began to change for the worse. Seeing no other choice, Stacy switched to a part‐time job, taking turns with another part‐time working libertarian mom caring for each other's children. The meeting, Stacy explained, was to discuss expanding their “little co‐op” into a venture offering an alternative childcare environment to traditional schooling. This co‐op will be run by the parents themselves and be limited to parents who share their libertarian mindset: “We don't want to police morality, so you have to choose who you let in,” she explained. Finally, Stacy added that the space itself will be registered as an activity center or a church to circumvent the state requirements for licensing and curriculum.

What is striking about this explanation is how diverse it is. First, Stacy related the need for an alternative daycare to a private problem. Her kids were unhappy in traditional daycares so, to promote their wellbeing, she searched for alternatives. This raised the practical problem of providing her children with alternative care while maintaining a job. Thus, the co‐op became a solution for both problems. However, once she finished presenting the personal and practical values of the co‐op, Stacy moved on to discuss its political value—it is based on a certain morality, and it skirts government regulations. Thus, Stacy presented the daycare as a solution to three different problems of distinct scopes: A private problem of her own kids' displeasure with traditional daycares, a practical problem for parents struggling to balance personal and professional life, and a political problem for libertarians looking to establish educational institutions that are non‐authoritarian and not subject to government control.

Interestingly, this inconsistent framing did not result in confusion. Instead, as the discussion unfolded, different members focused on different scopes, allowing Stacy to switch between them based on people's varying concerns and the problem they wanted the co‐op to solve. Thus, when one participant expressed her frustration over how difficult it is to find part‐time childcare, Stacy reassured her that the place they will rent has office space, allowing parents to combine work and childcare. And when another mother stressed that she and her husband “want to move away from traditional models of childcare” where children are separated from their parents, Stacy described the co‐op as a “community center” where libertarian parents are not only present but can learn new non‐coercive parenting techniques from each other.

In this way, Stacy could coordinate the design and prospective operation of her alternative childcare institution among members concerned with different problems. It allowed her to present the same practice as both a practical solution to people's daily problems and a radical political project that dodges the government and reimagines how children are raised. Such “scope switching” was common when FSers tried to coordinate and find common ground for their alternative practices. But scope switching was not only a way to reach agreements and satisfy people's varying desires. Sometimes, it was simply a way to allow the group to live with disagreement.

That was the case in a similar workshop exploring alternative parenting approaches during another camping event. The speaker, Catherine, argued against a “radical unschooling” method some libertarians subscribe to, which stands for succumbing unconditionally to the child's wants. This, she argued, is dangerous because kids cannot be trusted to choose what is good for them. Allowing kids to eat junk food, she illustrated, will cause them to grow up sick and depend on government healthcare programs, eventually making them “wards of the state.” An audience member interjected in protest, saying she had read a book about radical unschooling and believes Catherine does not do justice to the method. In response, Catherine offered another example, this time from her personal experience. She told how, following her family's eviction from their home, her daughter began wetting her bed. In her search for help, Catherine consulted a “radical unschooler” who advised her to let her daughter do as she pleased. If the child wets the bed, they suggested, Catherine should just let her do it because this is her way of expressing her needs. This, Catherine said, has spiraled her life into chaos. She recalled how she used to be able to breastfeed her younger son as they co‐slept but now had to wake up twice every night to change her daughter's sheets. The unschooler's advice, she explained, had made her life miserable.

By “downshifting” the discussion from a general one about libertarian parenting approaches to a personal one about her struggles with her own daughter, Catherine was able to defuse the tension in the workshop. So long as the discussion was concerned with the general problem of the proper way to raise free and independent children, members of the audience could challenge Catherine's reasoning with their own theories and knowledge. But once the discussion became focused on Catherine's personal difficulties and the solution that had worked for her, the debate became moot. Other participants no longer needed to agree with Catherine about proper solutions as the problem was no longer “what is the proper alternative to authoritative parenting,” but rather “what allowed Catherine to regain comfort in her life.” Once Catherine changed the scope of the problem, the discussion was no longer about solving society's broad institutional maladies but about immediate and specific solutions for Catherine and her family. This took the political edge off the group's disagreement and turned the discussion into an innocuous one about personal preferences.

In such scenes, when FSers tried to reach an agreement about practices that were not fully institutionalized, activists often switched their interpretive scope as they tried to solve or avoid disagreements. Importantly, this is not to say that their motivations somehow changed or that they appreciated the political and apolitical meanings of their practices equally. It is entirely plausible that Stacy only brought up her personal and practical struggles as a rhetorical technique to engage her audience and sway them toward her political agenda. And Catherine may still have thought that her approach to childrearing is the most ideologically sound. However, regardless of their inner, personal reasoning, both women demonstrated their ability to move between interpretive scopes tactfully and creatively to maintain concord, and facilitate smooth coordination in the group.

Thus, the same practice could have a political meaning for some participants and an a‐political meaning for others; the same practice could even gain and lose its political significance over the course of the interaction to defuse tensions or satisfy people's varying concerns. In this way, the relations between practices and their radical political meanings depended less on the practice itself and more on the challenges activists faced when trying to coordinate the practice and their collective effort to overcome these challenges. Furthermore, these efforts illustrate how the banalization of radical practices—the toning down of their political significance—played an important part in their resilience. What may seem like ideological inconsistency was, in fact, an expression of activists' pragmatic ability to coordinate across differences and maintain collaboration by responding to emerging challenges extemporaneously and in real‐time.

### 3.3 Staged Interactions: Suspending Free Spaces' Routines to Talk Ideology

In the FSP's free spaces, alternative radical practices became embedded in the daily circumstances and demands of members' daily lives, making their everyday experience of these practices increasingly entangled with practical difficulties or problems of personal comfort. However, occasionally, FSers shifted their focus toward broader concerns, tying their coordination efforts more strongly to deep political questions about their shared radical goals and image of an ideal political community. In such moments, problems that might otherwise be discussed in practical or personal terms became a topic for moral reflection about the group's political mission and utopian vision. Yet these moments usually required creating special interactional settings that interrupted free spaces' day‐to‐day routines. Therefore, such moments of collective ideological reflection were the exception, not the norm, in the movement's daily life.

In New Hampshire, such moments usually occurred within very particular settings of staged discussions, where the interaction itself was framed as a special event such as a public meeting, a formal speech, an organized debate, etc. These public discussions commonly carried a certain aura of formality and pomp that prompted participants to “upshift” their interpretations of emerging problems and justify their solution in terms of the political good that they promote rather than their practical or personal utility. In other words, compared to private discussions, backstage workshops, or training sessions, FSers treated these events as part of their movement's “frontstage” (Goffman 1959), where they performed not only as themselves but also as representatives of their movement as a whole. Here, discussions revolved more exclusively around questions of “How should a libertarian movement act?” and “What will a utopian libertarian society look like?” Often, these staged discussions were part of preplanned ceremonial events, like the group's yearly speechmaking competition. At other times, they were organized hastily in response to some unusual problem that could not be resolved by other, more casual means.

Thus, one such staged discussion took place during the FSP's annual summer camping festival in response to a widespread uproar following organizers' decision to kick two participants out. The ousting decision came after the two men entered a locked storage room on the campground and grabbed a poker table they wanted to use, apparently without permission from the campsite management. The festival organizers, fearing repercussions from the campsite owner, approached the men during their poker game and asked them to leave the festival. The men insisted that the table's owner had permitted them to take it, and the discussion ignited a heated debate around the poker table, which soon ballooned into a camp‐wide agitation. Hoping to rein in the outspreading turmoil, the organizers decided to summon a public meeting the following evening to “hash things out.”

The meeting was designed as a panel discussion, with three of the organizers on one side, the two culprits on the other, and a sixth person acting as moderator. One organizer, George, explained that they had summoned this gathering because there had been “an issue between two groups about property,” emphasizing that “as libertarians, we take property rights very seriously.” Sarah, another organizer, added that this event is an opportunity to “serve restorative justice.” The moderator took this cue to describe restorative justice as an oppositional approach to the government's punitive (and thereby flawed) form of justice. He argued that, in “our libertarian community” we want to prove that we can do justice ourselves without ever having to summon the police. George added that, if we want a world without a state, we need to know how to deal with offenses, and “this is what we did last night.” Finally, Sarah asserted that “we need to be the best representatives of our principles,” and someone taking something that is not theirs’ is a fundamental violation of these principles.

At the request of an audience member, the moderator then reviewed the events of the prior evening. He compared the storage room to a “security box” and the entry to a break‐in and argued that: “Here in this festival, we can't have any property rights violations in any form.” He then described how the organizing team finally agreed with the two perpetrators that they could stay and help clean up the campsite when the festival was over. He explained how this represents true restorative justice: it compensates the organizers, who are the victims here since they would have been held responsible if anything in the storage room went missing. Leah, the third organizer, explained that, although they initially instructed the men to leave, the organizing team then asked itself how they would have wanted to solve this matter had the state not existed. The decision to enact restorative justice, she said, provided them with a good simulation of how things would be in a stateless society. Sarah agreed. “This is what we teach our kids,” she concluded.

Although they were speaking to a relatively large audience, the speakers in this discussion did not switch between practical and political explanations to appeal to a variety of people. Instead, their interpretation of the problem and its chosen solution was consistently framed as part of the group's grand political project and radical vision of an ideal libertarian society. From the beginning, they related the problem to the basic principles of the movement (property rights), and they presented their solution as an opportunity to practice an alternative model of justice that not only related to the incident at hand but was also an opportunity for the group to exhibit its principles. Indeed, it was something they could teach their children.

Notably, nothing in the incident itself demanded such political framing. In fact, outside the public meeting, the organizers themselves discussed the incident in terms of the personal discomfort it caused them or the practical difficulties it could create, particularly vis‐à‐vis the campground's management. But within the staged interaction, the incident was framed exclusively as a political problem. Regardless of what their personal motivations and justifications for making their decision was, within the setting of the formal public discussion, organizers felt compelled to speak in the name of the group's common cause and appeal to members' shared ideological vision. What made this incident a political issue, turning an otherwise mundane conflict into an exercise in radical justice, was the situational context in which it was discussed.

In the same way that training sessions prompted activists to downshift their discussions and focus on practical questions, staged interactions prompted them to upshift the discussion and focus on ideological goals. Such staged meetings were not uncommon in the FSP. Members held occasional community meetings or public events, at which members gave speeches, debated group practices, or aired grievances. It was in these public discussions that activists displayed their radical political vision most clearly and the radical meanings of their practices became most explicit. However, these discussions also required special coordination efforts, such as orchestrating the arrival of many participants, securing a venue, or procuring sound‐amplifying equipment. For this reason, as others have also noted (Glass 2010), moments of ideological reflection tended to be the exception rather than the rule in the movement's free spaces, and they marked a pause in the everyday activities of the group. Nevertheless, it was in these discussions that activists debated what they imagined to be their movement's true essence and their practices' true meaning. There, they connected their practices to questions about “who we are” and “what we stand for,” tying their practices clearly and explicitly to their utopian vision and radical political goals.

## 4 Conclusion

The mainstreaming of the far‐right would probably not have been possible without the ability to normalize certain radical behaviors and worldviews and integrate them into people's everyday lives. As others have also noted (Luger 2022), radical meanings often emerge within otherwise mundane practices. This study shows how the emergence of radical meanings, as well as their fading, reflect people's ongoing attempts to coordinate practices and respond to the problems that emerge in the process. In this way, the toning down of ideological messaging allows certain radical practices to become banal, mundane activities that can be carried out over time.

This banalization is an integral part of the process of radicalization, as it allows people to partake in radical activity even without attributing any political meaning to it. Thus, the dynamic of upshifting and downshifting everyday practices' political significance may help explain how people who are not radicalized can collaborate with radicalized actors, join their spaces, adopt their practices, and become exposed to their messaging.

Moreover, the situational nature of these processes should also direct our attention to how different kinds of spaces facilitate different situational dynamics, thus playing varying roles in people's radicalization processes. Certain spaces (e.g., outdoor festivals) may offer more favorable conditions for staged interactions and explicit ideological reflection, while others (e.g., family households) may facilitate more banal, ongoing coordination of radical practices in ways that align with people's everyday lives. It is plausible that non‐radicalized actors would find it easier to collaborate with radicalized ones in banal spaces and activities, and that these spaces work as “gateways” through which people enter the movement before moving to other spaces and joining more radical activities. Future research could expand our understanding of different kinds of free spaces, and of how radicalization is facilitated by members' ongoing transitions between them. Those looking to combat the expansion of the radical far‐right may be particularly interested in learning how redirecting people from certain spaces (e.g., by providing appealing alternatives) may shape the trajectory of their radicalization processes.

As past research can reveal, the interplay between banal and explicit radicalism is not unique to the FSP. Blee (2003), for example, found that racist groups often use violence strategically as a political spectacle that helps deter enemies, attract recruits, and form collective identities. However, as violence becomes routinized, it stops invoking commentary and becomes a commonsense strategy of action. The notion of “banal radicalism” invites us to think about such processes as an integral part of movements' ongoing efforts to coordinate action among diverse groups. It directs our attention to the everyday mechanisms that allow radical, even violent practices to endure, and helps us imagine how they can be appropriately and effectively addressed.

## Ethics Statement

This research has received the University of Southern California IRB approval.

## Consent

All participants in this study have been informed about its goals and consented to take part in it.

## Conflicts of Interest

The author declares no conflicts of interest.

## Data Availability

The data that support the findings of this study are available on request from the corresponding author. The data are not publicly available due to privacy or ethical restrictions.

## Endnotes

^1^
My use of the term “banal radicalism” is inspired by Michel Billig's idea of “banal nationalism” (Billig 1995). Billig criticized nationalism studies' excessive focus on “‘hot’ nationalist passion.” Instead, he explored how nationalist ideology is reproduced through mundane practices which are not consciously noticed, and that “carry no propositional message for the ordinary citizen to receive passively or consciously argue against” (Billig 2009, 349). Therefore, “banal” here refers to everyday local habits of meaning‐making that render ideological practices and interpretations commonplace or taken‐for‐granted, rather than passionately argued and celebrated (Skey 2009).

^2^
This form of political action is sometimes referred to in the literature as “prefigurative politics.” The term, coined by Boggs (1977), represents social movements' attempts to model, in the present, the ideal society they wish to bring about. Similarly, some have used the terms “lifestyle politics” and “everyday politics” to describe the political meanings and significance attributed to mundane everyday activities (Giddens 1991; Yates 2015a). The differences between these concepts can be subtle, and their distinct uses facilitate a nuanced discussion in the literature on the relationship between politics, radicalism, and everyday life. However, such nuances are beyond the scope of this paper. To avoid conceptual overload, I grouped all these concepts under colloquial terms like “radical practices” or “alternative practices,” referring broadly to everyday practices people adopt as part of a radical political agenda.

^3^
Some theorists, like Mudde (2019) and Rydgren (2018) distinguish, within the far‐right, between the “extreme right,” which fully rejects the essence of democracy, and the “radical right,” which accepts democracy, but fundamentally challenges its key institutions and values. For simplicity's sake, and because the term “extremism” is sometime used to denote movements that condone violent action (see Futrell et al. 2018), I chose to use the term “radical far‐right” to capture all forms of right‐wing, anti‐liberal‐democracy movements.

^4^
This latter belief is often associated with antisemitic tropes about Jews being secretly in control of Western governments, sometimes referred to as the “Zionist Occupied Government” conspiracy theory, or ZOG (Simi and Futrell 2010). However, I did not encounter any mention of ZOG or references to a Jewish conspiracy during my time in the field. It is possible that people did not mention this theory to me because I am Jewish and made no effort to hide it. Then again, the FSP had several prominent and well‐appreciated Jewish members. So, if some members did subscribe to the ZOG conspiracy theory, this did not seem to direct the group's politics in any significant way.
